# MicroRNA-183-5p promotes the proliferation, invasion and metastasis of human pancreatic adenocarcinoma cells

**DOI:** 10.3892/ol.2020.12373

**Published:** 2020-12-14

**Authors:** Fei Miao, Jinhai Zhu, Yanlin Chen, Nanhong Tang, Xiaoqian Wang, Xiujin Li

Oncol Lett 11: 134-140, 2016; DOI: 10.3892/ol.2015.3872

Subsequently to the publication of this paper, an interested reader drew to the authors’ attention that a pair of data panels contained strikingly similar and overlapping data in Fig. 3D (specifically, the “NC” and “PANC-1” panels).

The authors have re-examined their data and realized that Fig. 3 was assembled incorrectly. The revised version of Fig. 3, containing the correct data for the “NC” group in Fig. 3D, is shown on the next page. The authors regret the inadvertent errors that were made during the preparation of the published figure, and confirm that these errors did not seriously affect the conclusions reported in the paper. The authors are grateful to the editor of *Oncology Letters* for allowing them the opportunity to publish a Corrigendum, and all the authors agree to this Corrigendum. Furthermore, they apologise to the readership for any inconvenience caused.

## Figures and Tables

**Figure 3.**
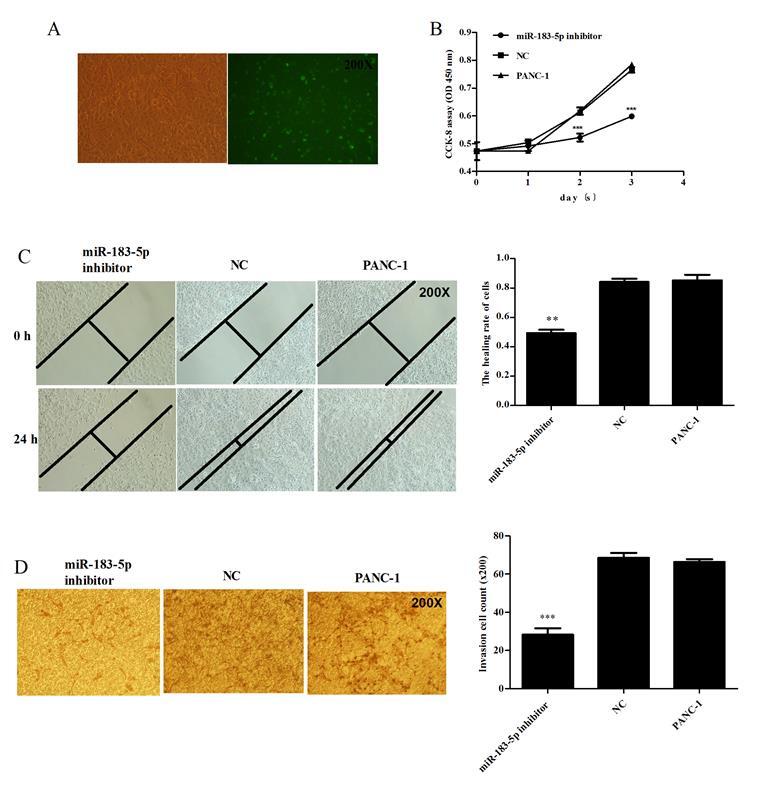
(A) miR-183-5p transfected PANC-1 pancreatic carcinoma cells; the transfection efficiency was ~60%. (B) CCK-8 assay: Cell proliferation decreased significantly in PANC-1 cells transfected with miR-183-5p inhibitor compared with PANC-1 cells transfected with NC (P<0.001) and untreated PANC-1 cells (P≤0.001). (C) Wound-healing assay: Adhered cell monolayers were scratched with a 20 µl pipette tip and wound-healing capacity was monitored by microscope after 0 and 24 h. The wound closure rate of PANC-1 cells transfected with miR-183 inhibitor was significantly lower than that of PANC-1 cells transfected with NC (P=0.0004) and untransfected PANC-1 cells (P=0.0012). (D) PANC-1 cells were transfected with miR-183-5p inhibitor or NC for 48 h; cell invasion ability was measured by Matrigel invasion assays. After transfection with miR-183-5p inhibitor, the PANC-1 cell invasion ability was lower compared with that of untransfected PANC-1 cells (P<0.0001) or cells transfected with NC (P<0.0001). miR, microRNA; NC, negative control; CCK-8, cell counting kit-8.

